# Quantification of tumor microenvironment acidity in glioblastoma using principal component analysis of dynamic susceptibility contrast enhanced MR imaging

**DOI:** 10.1038/s41598-021-94560-3

**Published:** 2021-07-22

**Authors:** Hamed Akbari, Anahita Fathi Kazerooni, Jeffrey B. Ware, Elizabeth Mamourian, Hannah Anderson, Samantha Guiry, Chiharu Sako, Catalina Raymond, Jingwen Yao, Steven Brem, Donald M. O’Rourke, Arati S. Desai, Stephen J. Bagley, Benjamin M. Ellingson, Christos Davatzikos, Ali Nabavizadeh

**Affiliations:** 1 Department of Radiology, Perelman School of Medicine, Hospital of University of Pennsylvania, University of Pennsylvania, Philadelphia, PA USA; 2 Center for Biomedical Image Computing and Analytics, Perelman School of Medicine, University of Pennsylvania, Philadelphia, PA USA; 3 UCLA Brain Tumor Imaging Laboratory, Center for Computer Vision and Imaging Biomarkers, David Geffen School of Medicine, University of California Los Angeles, Los Angeles, CA USA; 4 Department of Radiological Sciences, David Geffen School of Medicine, University of California Los Angeles, Los Angeles, CA USA; 5 Department of Neurosurgery, Perelman School of Medicine, University of Pennsylvania, Philadelphia, PA USA; 6 Division of Hematology-Oncology, Department of Medicine, Perelman School of Medicine, University of Pennsylvania, Philadelphia, PA USA

**Keywords:** Cancer imaging, CNS cancer

## Abstract

Glioblastoma (GBM) has high metabolic demands, which can lead to acidification of the tumor microenvironment. We hypothesize that a machine learning model built on temporal principal component analysis (PCA) of dynamic susceptibility contrast-enhanced (DSC) perfusion MRI can be used to estimate tumor acidity in GBM, as estimated by pH-sensitive amine chemical exchange saturation transfer echo-planar imaging (CEST-EPI). We analyzed 78 MRI scans in 32 treatment naïve and post-treatment GBM patients. All patients were imaged with DSC-MRI, and pH-weighting that was quantified from CEST-EPI estimation of the magnetization transfer ratio asymmetry (MTR_asym_) at 3 ppm. Enhancing tumor (ET), non-enhancing core (NC), and peritumoral T2 hyperintensity (namely, edema, ED) were used to extract principal components (PCs) and to build support vector machines regression (SVR) models to predict MTR_asym_ values using PCs. Our predicted map correlated with MTR_asym_ values with Spearman’s *r* equal to 0.66, 0.47, 0.67, 0.71, in NC, ET, ED, and overall, respectively (*p* < 0.006). The results of this study demonstrates that PCA analysis of DSC imaging data can provide information about tumor pH in GBM patients, with the strongest association within the peritumoral regions.

## Introduction

Glioblastoma (GBM) is the most common malignant primary brain tumor in adults, characterized with vasular proliferation, diffuse infiltration in the adjacent brain parenchyma, and resistance to the standard therapies^1^. The tumor microenvironment plays an important role in abundant infiltration of GBM tumor cells, its resistance to standard therapies, recurrence and therefore, poor patient prognosis^2^. Due to rapid growth of GBM tumors and actively migrating cell population, hypercellular regions are formed typically surrounding the necrotic foci tissues and have a high metabolic demand^3^. When the tumor grows, the lack of sufficient circulation compared to the cell population of the tumor results in ischemia and secretion of angiogenic factors, which in turn leads to proliferation of new vessels^4^.

Neo-angiogenesis forms a tortuous and branched vascular structure with increased blood volume and permeability, and impaired cerebral perfusion with subsequent necrosis^3,5^. These alterations promote tumor growth, decrease oxygen, increase glycolysis and lactic acid, decrease extracellular pH, facilitate cell invasion^6^. This augments the probability of mutations, such as vascular endothelial growth factor (VEGF) gene expression triggered by the hypoxia-inducible factor (HIF) family of transcription factors^7,8^. Even in presence of abundant oxygen, glycolysis is often enhanced in cancers due to elevated concentration of lactic acid, resulting in a substantial decrease in extracellular pH which leads to escalated invasion and aggressiveness of the tumor, and decreased immune response^6,9,10^.

Various MRI techniques have been used to measure tumor PH. Magnetic resonance spectroscopy (MRS) reveals the amount and spatial distribution of particular biochemical substances involved in metabolic processes in tumoral tissues^11^. ^1^H MRS, ^13^C MRS, and phosphorus MRS (^31^PMRS) can estimate levels of cellular metabolites and tumoral pH^11,12^. Chemical exchange saturation transfer (CEST) is a relatively new MR technique that images certain compounds at very low concentrations that are too low to be directly detected by MRS technique^13^. Tumor blood perfusion may also reflect the underlying tumor pH, as well-perfused tumors have more efficient removal of excess lactic acid, protons, CO_2_, and other metabolic byproducts. Dynamic susceptibility contrast (DSC) perfusion is a widespread clinical MRI approach for quantification of cerebral perfusion and is often collected for assessment of brain tumors^14^. As DSC-MRI can measure tissue perfusion and compromised microvasculature in GBMs, it might be able to quantify tumor acidity.

Amine CEST echo-planar imaging (CEST-EPI) is a fast molecular imaging MRI technique to measure tumor pH^6^. In a recent study, a positive linear correlation between cerebral blood volume (CBV) obtained from DSC perfusion MRI and acidity was demonstrated in areas of T2-hyperintense, non-enhancing tumor in glioma patients^15^. We hypothesize that principal component analysis (PCA) of DSC-MRI perfusion images in conjunction with machine learning (ML) techniques in patients with GBM may quantify microvascular structure at the voxel level and infer capillary-level hemodynamics that correlates with tumor acidity. PC analysis of DSC-MRI has shown potential in predicting the location of future recurrence^16,17^, patient’s survival ^18^, arteriovenous shunting^4^, and *EGFRvIII* status^19^. The aim of this analysis is to use ML methods based on perfusion MRI scans to uncover unique tissue characteristics that correlate with tissue acidity and might provide insights about the tumoral and peritumoral tissue metabolism to guide treatment planning.

## Results

In this prospective study, we included 32 patients (19 males, 13 females; age, 64.6 ± 10.11 years old), who were confirmed to have GBM tumors (Table 1). A total of 101 CEST-MRI scans were acquired from the study subjects (12 patients had pre-surgical and 89 had imaging during active treatment with radiation and/or chemotherapy), of which, 78 scans with their corresponding DSC-MRI scans passed our data preparation and pre-processing steps. The SVR machine learning method based on PCA was applied in a cohort of 78 cases.

**Table 1:** Patient demographics.

Total no. of patients	32
Pre-surgery timepoints	12
Post-surgery timepoints	89
**Age (years)**	
Mean	64.6 ± 10.11
Median	66.5
Range	40
**Sex**	
Male	19
Female	13
***IDH* status**	
Wild-type	29
Mutant	3
***MGMT-*promoter methylation status**	
Methylated	20
Unmethylated	12

**Figure 1:**
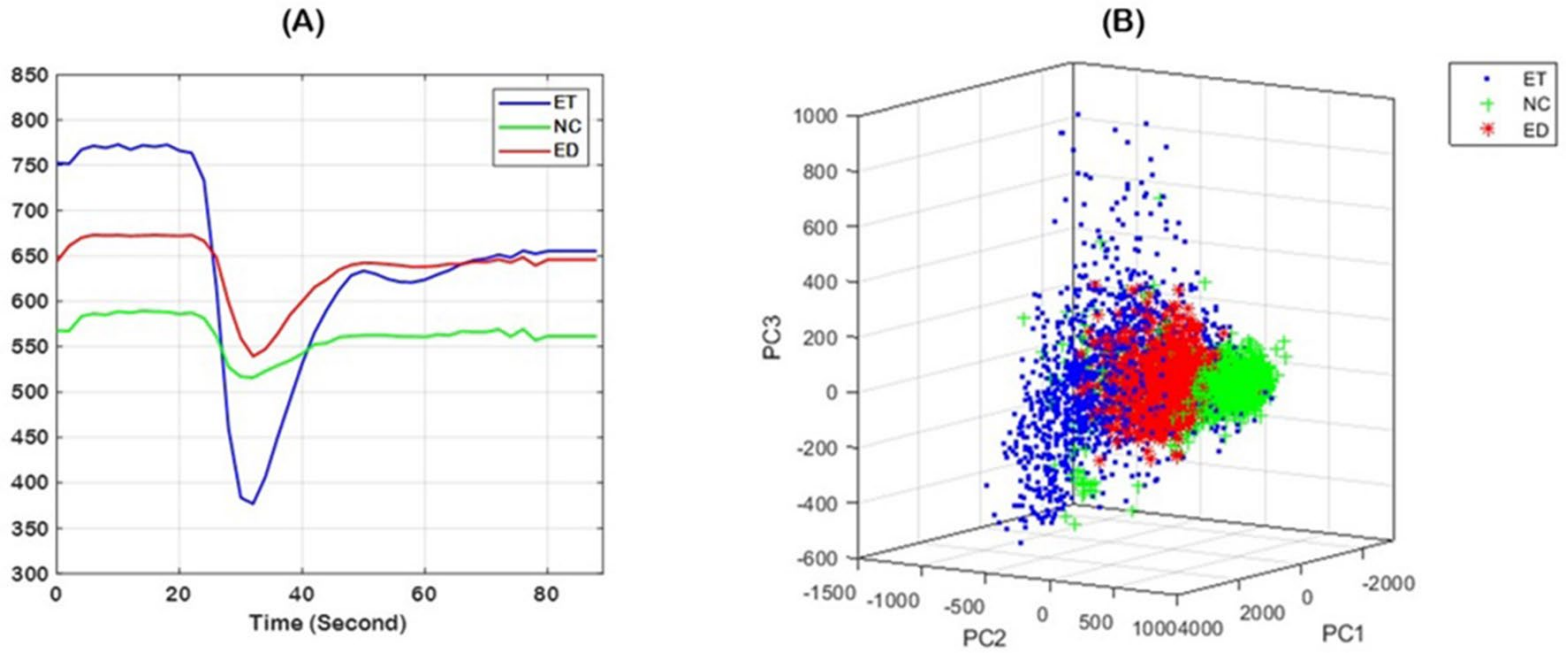
An illustration of the perfusion time-series in tumorous subregions, i.e., ET, NC, and ED (**A**); and the clustering of each tissue type using PC analysis (**B**), signifying the potential of the PCs in capturing tissue characteristics. PC1, PC2, and PC3 represent the first, second, and third principal components, respectively. *ET* Enhancing tumor, *NC* Necrotic core, *ED* paeritumoral edema.

**Figure 2:**
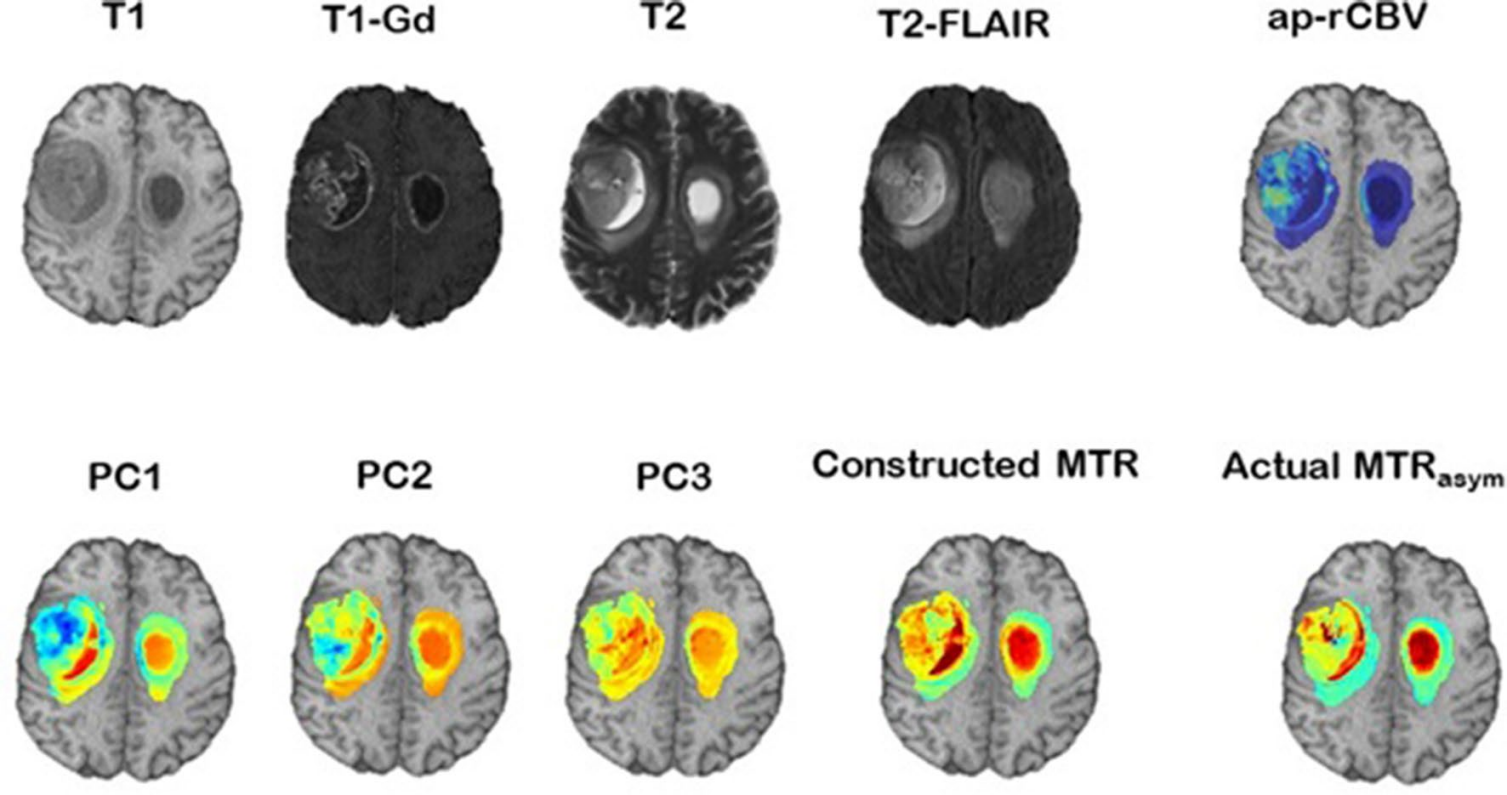
Conventional MRI, including T1, T1-Gd, T2, and T2-FLAIR, scans of a 58-year-old male patient included in our study. Map of a proxy to relative cerebral blood volume (ap-rCBV) derived from DSC-MRI scans with CaPTk software. Three principal components (PCs), PC1 to PC3, calculated using PCA of the hemodynamic perfusion curves, along with the MTR_asym_ image constructed using the seven PCs in association with the actual MTR_asym_ image. CaPTk version 1.8.1 (www.med.upenn.edu/cbica/captk/).

Principal component analysis of perfusion time-series revealed that the tumor subregions, i.e., ET, NC, ED, form characteristic clusters (Fig. 1B), which facilitate specification of the tissues and allow for mapping the heterogeneity within a specific tissue. Figure 2 illustrates structural MRI scans, including T1, T1-Gd, T2, T2-FLAIR images for a male patient (58 years old) with GBM. Furthermore, relative cerebral blood volume (rCBV) map generated from DSC-MRI scans using CaPTk software, PC1–PC3 images derived from PC analysis of the hemodynamic curves, the MTR_asym_ image constructed using our proposed approach, along with the actual MTR_asym_ image quantified from CEST imaging are shown in the whole pathogenic region. As it can be inferred, our constructed MTR_asym_ provides more accurate voxel-wise mapping of the actual MTR_asym_ image, compared to the rCBV map. Among the seven PCs used in building our model, the visual similarity is most striking for the first three PC images since the components progressively capture less variance.

**Figure 3:**
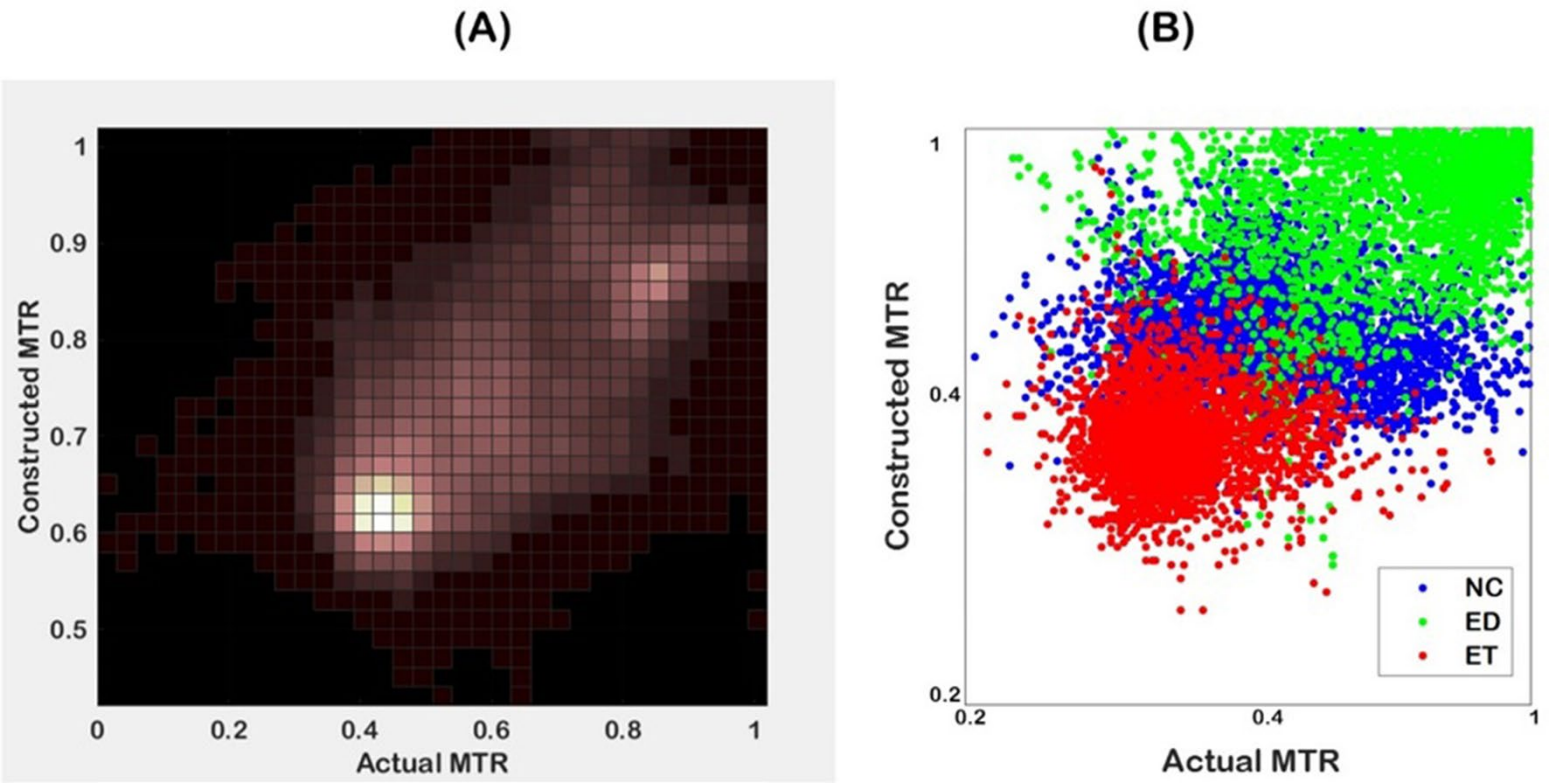
Demonstration of (**A**) bivariate histogram of the constructed in comparison with actual MTR_asym_ images; and (**B**) association of the clusters of tumor tissues in the constructed versus actual MTR_asym_ image.

The MTR_asym_ image constructed from perfusion PCs using our proposed regression method showed moderate to strong agreement with the MTR_asym_ image, with R of 0.47 (*p* = 0.006), 0.66 (*p* = 0.0009), 0.67 (*p* = 0.00001) in the ET, NC, and ED regions, respectively, and 0.71 (*p* < 10e-6) in the whole pathogenic region, as a union of ET, NC, and ED areas, averaged over all the patients. Figure 3 demonstrates a strong association of the MTR_asym_ image built from the perfusion PCs and the actual MTR_asym_ image, implying the potential of ML in distinguishing the tumorous regions with specific metabolism characteristics.

**Figure 4:**
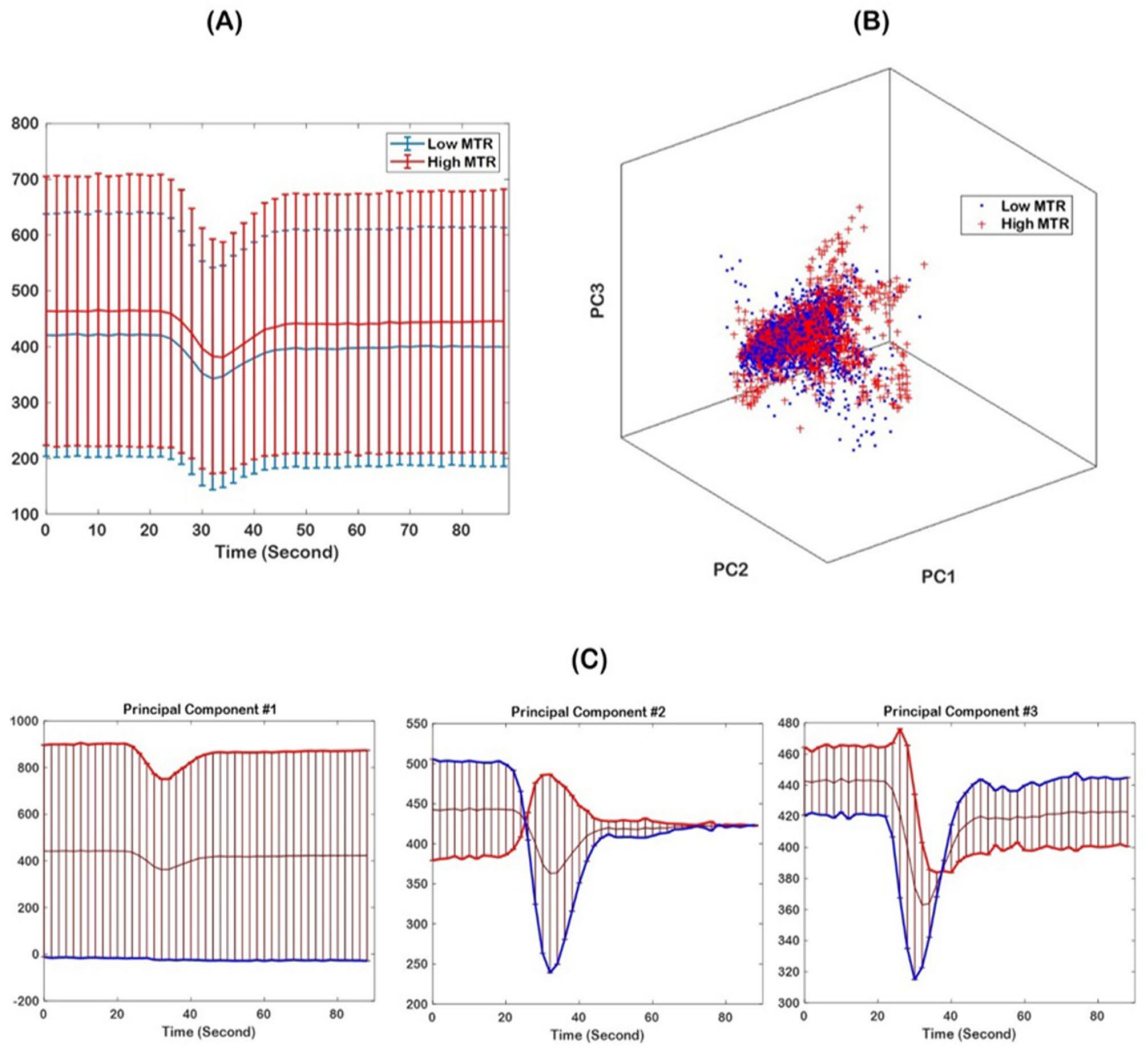
(**A**) Perfusion curves calculated within regions of low and high MTR_asym_ (shown in blue and red colors, respectively), suggesting poor discrimination of the regions solely based on hemodynamic curves. (**B**) Discrimination of low and high MTR_asym_ regions based on PC analysis; PC1 = principal component 1; PC2 = principal component 2; PC3 = principal component 3. (**C**) The three principal components for high MTR_asym_ regions, yielding a marked differentiation of these regions based on the PCs.

**Figure 5:**
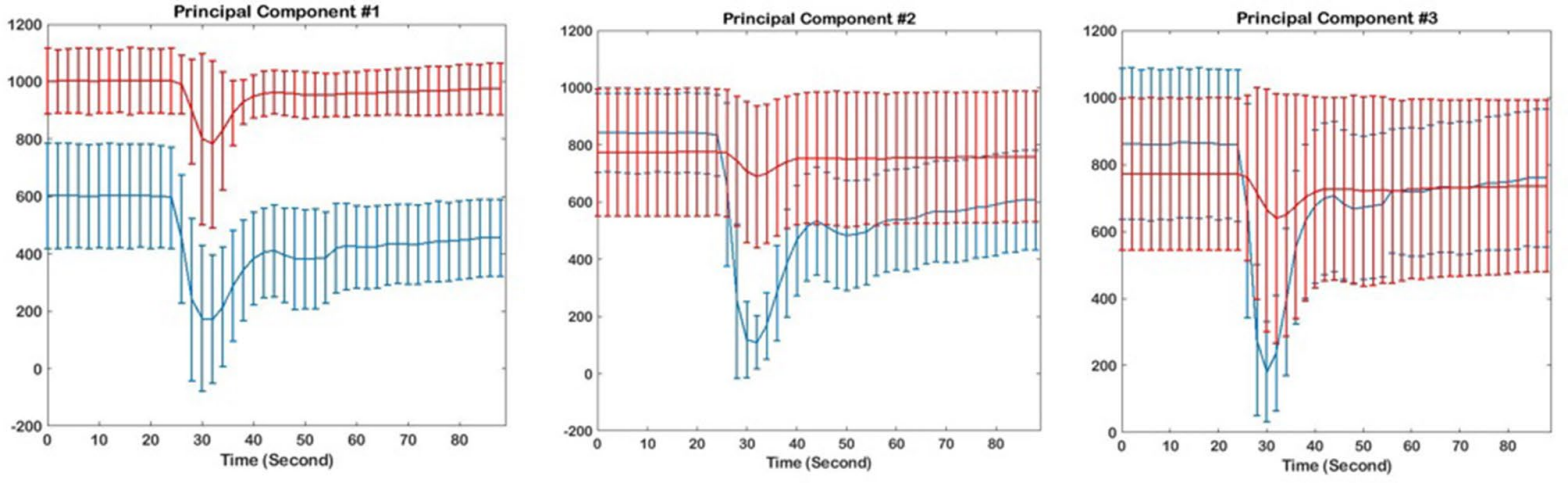
The perfusion curves calculated form the regions with highest (red) and lowest (blue) values on individual Principal Component images: (left) Principal Component 1; (middle) Principal Component 2; and (right) Principal Component 3.

This method outperforms the conventional DSC-MRI analysis, as displayed in Fig. 4, where panel (A) presents the signal intensity–time curves of the voxels located in the high (red) and low (blue) MTR_asym_ image. While the voxels have been selected from the regions with different levels of acidic pH, the perfusion curves are not discriminant. Applying PC analysis differentiates the clusters of the same high and low MTR_asym_, as it can be observed from panel (B) of Fig. 4. The bottom panel (part C) shows three PCs quantified for the higher and lower MTR_asym_ regions. Figure 4C reveals that the first principal component (PC1) is primarily related to the level of the perfusion signal, as evidenced by the large variance throughout the signal time course. The second principal component (PC2) is related to the depth of the perfusion signal drop, in relation to the baseline level, and the third principal component (PC3) relates to the shape of the drop of the perfusion signal, e.g., steepness of the signal drop and recovery. As a comparison, Fig. 5 displays the signal intensity–time curves in the highest (red) and lowest (blue) voxels in PC1, PC2, and PC3 images, suggesting the differentiability of the tissues based on PC analysis. Specifically, PC1 provides a noticeable discrimination between the areas with different hemodynamic properties. The discrimination diminishes in larger PCs as evidenced by this illustration.

## Discussion

Our study showed that high‐resolution pH‐sensitive imaging in brain tumors can be achieved on clinical 3T MR systems using DSC-MRI with strong correlation to CEST-EPI PH imaging. DSC-MRI can characterize microvascular circulation in GBM patients, and the respective acidity can be extracted using PCA and ML techniques. The proposed techniques are ideally suited for evaluation of malignant gliomas since GBM remains the most angiogenic primary brain tumor and therefore exhibits extensive neovascularization, compromised brain blood barrier, and heterogeneous acidity. DSC-MRI without proper processing cannot discern the pH heterogeneity of the tumoral regions, but as proposed in this study, PCA of the perfusion time-series can be used to differentiate tissue acidity.

GBMs have unique pathophysiological characteristics such as significant invasiveness, fast growth, and rapid seeding. Also, similar to the other cancers, GBMs prefer glycolysis over oxidative phosphorylation even in the presence of ample oxygen (Warburg effect) that results in increased intracellular lactate and elevated acidification. These tumors also cause direct destruction of surrounding tissue, including both neuronal death via glutamate excitotoxicity^20^ and degradation of the extracellular matrix via metalloproteinases and other proteases^21^ that are pH-dependent^22,23^. Since, ion movement is directly coupled to movement of other ions, pH not only serves as a regulator of cell activity, but also an indirect surrogate marker of other cellular functions. PH heterogeneity in the tumor microenvironment is critical for surgical and chemo-radiation planning. Weak base and weak acidic drugs get trapped in either the intracellular or extracellular spaces due to “ion trapping” phenomena^24^.

Wang et al. investigated association between pH-sensitive CEST-EPI and relative cerebral blood volume (CBV) measurements obtained from DSC-MRI in patients with gliomas^15^. They reported a strong correlation between acidity and CBV in the T2-hyperintense regions, but not in the areas of enhancing tumor. In agreement with this study, our results demonstrated that the correlation between our constructed MTR_asym_ map and the actual MTR_asym_, as quantified by CEST-EPI, is highest in the peritumoral T2-hyperintense areas, followed by necrotic tumor core, and contrast-enhancing tumor regions. However, in our study, in different subregions of each tumor, a heterogeneous distribution of high-acidity tissues was observed.

The proposed PCA-derived features integrated with SVR method uses temporal dynamics of DSC-MR imaging. Our results indicate that a more comprehensive analysis of DSC along with machine learning, can unravel useful information related to tumor acidity, which is typically not obtained with traditional CBV or CBF maps. Specifically, higher acidotic tissues demonstrate higher level of DSC signal based on PC1, consistent with higher necrosis and free water mobility. PC2 mainly represents the depth of drop in DSC signal, and deeper drop in the signal shows higher acidity. Integrative information of signal drop and recovery is stored in PC3, where lower signal recovery is associated with higher acidotic tissues.

CEST-EPI has been shown to be effective an a non-invasive biomarker to distinguish between IDH1 mutant and wild type gliomas, and also 1p/19q codeleted from intact IDH1 mutant gliomas^25,26^. In addition, it has been shown to be of value as an early imaging biomarker for bevacizumab treatment response and failure in recurrent glioblastoma^27^. Our proposed approach could support measuring tumor acidity with DSC-MRI, as a more widely-accessible imaging method compared with CEST-EPI.

There are limitations to our study, including limited sample size and single institutional data collection; these results need to be validated in a large multi-institutional study.

In conclusion, the results of this study indicate that PCA analysis of DSC-MRI in conjunction with machine learning techniques, can potentially enable better localization of highly acidic regions. In turn, this information may be used for tumor prognostication and treatment response evaluation.

## Methods

### Patients

Institutional review board (IRB) approval of the University of Pennsylvania was obtained for this prospective study and informed consent was collected from the participants. All methods were carried out in accordance with relevant guidelines and regulations. 32 subjects with intra-axial brain mass suggestive of high-grade glioma, who were referred to the radiology department of hospital of University of Pennsylvania from March 2018 to February 2020 and were subsequently proven by histopathology to be GBM were included in this study.

### Image acquisition

All MRI scans were performed on a Magnetom Tim Trio 3 Tesla scanner (Siemens, Erlangen, Germany) using a 12-channel phased array head coil. Conventional MRI sequences included axial T1-weighted (T1) before and after administration of gadolinium contrast agent (T1-Gd) with matrix size = 192 × 256 × 192, resolution = 0.98 × 0.98 × 1.00 mm^3^, repetition time (TR in ms)/echo time (TE in ms) = 1760/3.1, T2-weighted (T2) with matrix size = 208 × 256 × 64, resolution = 0.94 × 0.94 × 3.00, TR/TE = 4680/85; T2 fluid-attenuated inversion recovery (T2-FLAIR) with matrix size = 192 × 256 × 60, resolution = 0.94 × 0.94 × 3.00, TR/TE = 9420/141.

DSC-MR imaging was performed by a gradient-echo echo-planar (GE-EPI) imaging sequence during a second 0.1-mmol/kg bolus of Dotarem (Gadoterate Meglumine) with the following parameters: TR/TE = 2000/45 ms, FOV = 22 × 22 cm^2^, resolution = 1.72 × 1.72 × 3 mm^3^, 20 sections. A bolus of contrast agent with a dose of 0.1 mmol/kg which was done for DCE (dynamic-contrast-enhanced) imaging served as a preload dose for DSC imaging to reduce the effect of contrast agent leakage on relative cerebral blood volume (rCBV) measurements.

Acquisition of pH‐sensitive information was performed through an amine contrast specific for single-echo CEST-EPI sequence^15,28^. MR imaging acquisition parameters included the following: FOV = 240–256 × 217–256 mm, matrix size = 128 × 116–128, slice thickness = 4 mm with no inter-slice gap, 25 consecutive slices, excitation pulse flip angle = 90°, TE = 27 ms, bandwidth = 1628 Hz, and generalized auto-calibrating partially parallel acquisition factor = 2–3. Off-resonance saturation was applied using a pulse train of 3 × 100 ms Gaussian pulses with a peak amplitude of 6 microtesla. A total of 29 off-resonance or z-spectral points were sampled at frequency offsets of − 3.5 to − 2.5 ppm, − 0.3 to + 0.3 ppm, and − 2.5 to + 3.5 ppm, all in increments of 0.1 ppm. A reference scan (S0) was obtained with the same acquisition parameters, without the saturation pulses. Total scan time for CEST-EPI was approximately 6 min.

### MRI pre-processing

For each patient, all MRI volumes (T1, T2, T2-FLAIR, DSC-MRI and MTR_asym_) were rigidly co-registered with their corresponding T1-Gd using the Greedy registration method^29^ (https://github.com/pyushkevich/greedy). Subsequently, all conventional MRI scans (T1, T1-Gd, T2, T2-FLAIR) were smoothened to remove any high frequency intensity variations (i.e., noise)^30^, corrected for magnetic field inhomogeneities using N4ITK method^31^ and skull-stripped using FSL BET^32^ followed by manual revision when needed. For brain tumor segmentation in the images, DeepMedic^33^, a Deep Learning (DL)-based segmentation algorithm in Cancer Imaging Phenomics Toolkit (CaPTk) v.1.7.8^34,35^ which had been trained on BraTS 2017 training data, was applied to the co-registered conventional MRI scans. Brain tumor segmentation delineated three regions of interest (ROIs), i.e., enhancing tumor (ET), necrosis (NC), and peritumoral edema (ED), in the GBM tumors.

### Amine CEST-EPI post-processing

Clinical post-processing of CEST-EPI consisted of affine motion correction (MCFLIRT; FSL, https://fsl.fmrib.ox.ac.uk/fsl/fslwiki/MCFLIRT) and B0 correction via a z-spectra-based K-means clustering and Lorentzian fitting algorithm^36^. An integral of the width of 0.4 ppm was then obtained around both the − 3.0 and + 3.0 ppm (− 3.2 to − 2.8 and + 2.8 to + 3.2 ppm, respectively) spectral points of the inhomogeneity-corrected data. These data points were combined with the S0 image to calculate the asymmetry in the magnetization transfer ratio (MTR_asym_) at 3.0 ppm as defined by equation MTR_asym_ (ω) = S(− ω) − S(ω)/S_,_ where ω is the offset frequency of interest (3.0 ppm). All resulting maps were registered to high-resolution post-contrast T1-weighted images for subsequent analyses.

### Temporal principal component analysis

Principal component analysis (PCA) is a dimensionality reduction method^17^ which was used in this study to distill the DSC-MRI time series down to a few components that capture the temporal dynamics of blood perfusion. All hemodynamic perfusion curves were aligned and normalized for the baseline and maximum drop across the patients^37^. We randomly selected voxels in each tumor subregion, i.e., ET, NC, and ED, and generated their signal intensity–time curves (Fig. 1A). PCA was subsequently applied to capture the variance of the time series in all the ROIs and all subjects. Because of the relative consistency in the perfusion patterns of the various ROIs, seven principal components were sufficient to capture more than 99% of the variance in the perfusion signal for all tumor subregions and all patients.

### Generation of MTR_***asym***_ images based on PCs using machine learning

We built several regression models for tumor subregions using support vector machine regression (SVR) aiming to predict the MTR_asym_ values from the seven PCs on a voxel-by-voxel basis to create a PC-derived MTR_asym_ image, referred to as constructed MTR_asym_ image. Leave-one-subject-out cross-validation of these predictive models was performed to ensure that the model and the associate estimates of accuracy would likely generalize to new patients. We trained the SVR models separately in ET, NC, and ED regions using Gaussian kernel functions with an automatic kernel scale and sequential minimal optimization (SMO) configuration. Performance of the SVR method was evaluated using Spearman’s correlation. All machine learning and statistical analyses was performed in MATLAB 9.4.0.949201 (R2018a) Update 6.
